# Comparison of the Photobleaching and Photostability Traits of Alexa Fluor 568- and Fluorescein Isothiocyanate- conjugated Antibody

**Published:** 2011-09-23

**Authors:** Jafar Mahmoudian, Reza Hadavi, Mahmood Jeddi-Tehrani, Ahmad Reza Mahmoudi, Ali Ahmad Bayat, Elham Shaban, Mohtaram Vafakhah, Maryam Darzi, Majid Tarahomi, Roya Ghods

**Affiliations:** 1. Department of Immunochemistry, Monoclonal Antibody Research Center, Avicenna Research Institute, ACECR, Tehran, Iran; 2. Department of Antibody-Antigen Engineering, Monoclonal Antibody Research Center, Avicenna Research Institute, ACECR, Tehran, Iran; 3. Division of Clinical Immunology, Department of Laboratory Medicine, Karolinska Institute at Karolinska University Hospital Huddinge SE-141 86 Stockholm, Sweden; 4. Reproductive Biotechnology Research Center, Avicenna Research Institute, ACECR, Tehran, Iran

**Keywords:** Fluorescein Isothiocyanate, Alexa Fluor 568, Photostability, Photobleaching

## Abstract

**Objective:**

Synthetic fluorescent dyes that are conjugated to antibodies are useful tools to probe molecules. Based on dye chemical structures, their photobleaching and photostability indices are quite diverse. It is generally believed that among different fluorescent dyes, Alexa Fluor family has greater photostability than traditional dyes like fluorescein isothiocyanate (FITC) and Cy5. Alexa Fluor 568 is a member of Alexa Fluor family presumed to have superior photostability and photobleahing profiles than FITC.

**Materials and Methods:**

In this experimental study, we conjugated Alexa Fluor 568 and FITC dyes to a mouse anti-human nestin monoclonal antibody (ANM) to acquire their photobleaching profiles and photostability indices. Then, the fluorophore/antibody ratios were calculated using a spectrophotometer. The photobleaching profiles and photostability indices of conjugated antibodies were subsequently studied by immunocytochemistry (ICC). Samples were continuously illuminated and digital images acquired under a fluorescent microscope. Data were processed by ImageJ software.

**Results:**

Alexa Fluor 568 has a brighter fluorescence and higher photostability than FITC.

**Conclusion:**

Alexa Fluor 568 is a capable dye to use in photostaining techniques and it has a longer photostability when compared to FITC.

## Introduction

 During the last decades bioconjugation of synthetic fluorescent dyes has provided valuable tools for histochemical and cytochemical research (1, 2). Photostable and brighter dyes are useful tools to apply in photostaining techniques. To this end,the comparison of dye physicochemical characteristics such as photobleaching and photostability is a valuable way to identify the best dyes (2-8).

 Based on the chemical structure of dyes, their photostability and photobleaching profiles are very different. The Alexa Fluor dyes contain superior fluorophores with fluorescent emissions that span the visible spectrum and beyond. Their photostable characteristic permits capturing images that were previously unattainable with conventional fluorophores such as fluorescein isothiocyanate (FITC).

 It is believed that generally Alexa Fluor dyes have brighter fluorescence and more photostability than FITC (8). Alexa Fluor 568, a member of Alexa Fluor family, absorbs light at 578 nm and emits at 603 nm (8) while FITC absorbs at 495 nm and emits at 521 nm (9). In the present study, Alexa Fluor 568 and FITC were conjugated to a mouse anti-human nestin monoclonal antibody (ANM); subsequently, the number of fluorophores (dyes) per protein (antibody molecule) was calculated.

 Finally, we examined their functionality, long scale fluorescence, and photostability by microscopic analysis of immunocytochemistry (ICC) stained cell spreads.

## Materials and Methods

### Conjugation of Alexa Fluor 568 and FITC to ANM

 In order to make Alexa Fluor 568 conjugate, ANM (clone 4G10G8, IgG) prepared at Avicenna Research Institute (Tehran, Iran) (10) was dialyzed against bicarbonate buffer (0.1 M; pH= 8.3) overnight at 4℃. Alexa Fluor 568 (Invitrogen, California, USA) was dissolved in DMSO. A total of 90 µg Alexa Fluor 568 was mixed with 1 mg ANM in a total volume of 1 ml. After one hour mixing at room temperature (RT) the mixture was dialyzed against Phosphate buffered saline (PBS) over night at 4℃ (11).

 Also in FITC conjugate, ANM was dialyzed against bicarbonate buffer (0.1 M; pH= 8.3) overnight at 4℃. FITC (Sigma-Aldrich, Wisconsin, USA) was dissolved in dimethyl sulfoxide (DMSO) subsequently FITC (20 µg) was mixed with ANM (1 mg) in a total volume of 1 ml. After mixing for one hour at RT the mixture was dialyzed against PBS over night at 4℃ (9).

### Determination of degree of labeling (DOL)

 Fluorophore/antibody ratios were determined three times by measuring the absorbance of the antibodies at 280 nm and the absorbance of the dyes at their maximum excitation wavelength (λ max)with the following formula: DOL = Amax × MW / [antibody] × єdye
Where Amax = absorbance of dye molecules in λ max; MW = the molecular weight of the antibody;[antibody] = antibody concentration (mg/ml); and єdye = the extinction coefficient of the dye at its maximum absorbance (12).

### Immunocytochemical staining

 A total of 20000 bovine sertoli cells (BSC)(10) were cultured in RPMI 1640 medium that contained 10% (v/v) fetal calf serum (Invitrogen,California, USA) and 1% penicillin/streptomycin (Sigma-Aldrich) at 37℃ in the presence of 5% CO_2_ on glass slides, followed by acetone fixation. After washing, cells were blocked with 5% mouse serum; subsequently, Alexa Fluor 568- and FITC- labeled ANM (1 mg/ml, dilution: 1/100) were added followed by incubation for 1 hour at RT. Cells were then washed with PBS and directly observed under a fluorescent microscope (Olympus, Tokyo, Japan). This procedure was repeated three times.

### Photobleaching analysis

 Samples were continuously illuminated and digital images acquired under a fluorescent microscope. We saved images every 5, 20, 30, 40 and 80 seconds. Digital images taken every 5 and 30 seconds were processed by ImageJ 1.421 software (Wayne Rasband, National Institutes of Health, Bethesda, MD, USA).

### Ethical consideration

 All procedures were conducted according to the guidelines of the Animal Care and Ethics Committee of Avicenna Research Institute.

## Results

### Determination of fluorophore/antibody ratios

To determine the conjugation quality, fluorophore/antibody ratios were calculated using the DOL formula. By using the DOL formula, we concluded that 9 moles of Alexa Fluor 568 and 7 moles of FITC were conjugated to each mole of ANM.

### Photobleaching of FITC- and Alexa Fluor 568- conjugated antibodies

**Fig 1:**
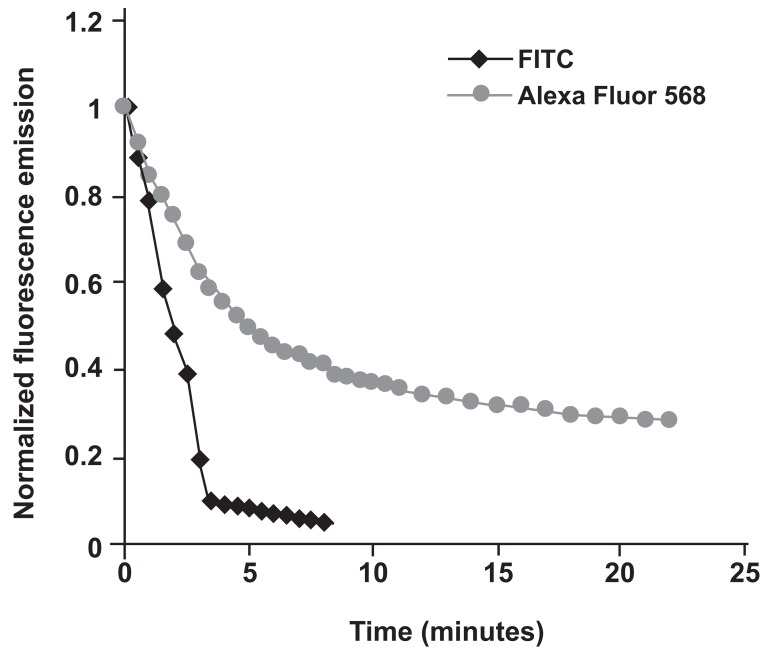
Comparison of long-scale fluorescent profiles of the FITC- and Alexa Fluor 568- conjugated ANM. Images were captured every 30 seconds followed by analysis of the data by ImageJ software.

 To define the long scale fluorescence of FITC- and Alexa Fluor 568- conjugated ANM, their normalized fluorescence emission (13) provided by ImageJ software were plotted against time by using ICC stained sertoli cells (Fig 1). According to figure 1, FITC lost its brightness earlier than Alexa Fluor 568.

### Analysis of photostability of FITC- and Alexa Fluor 568- conjugated ANM

**Fig 2:**
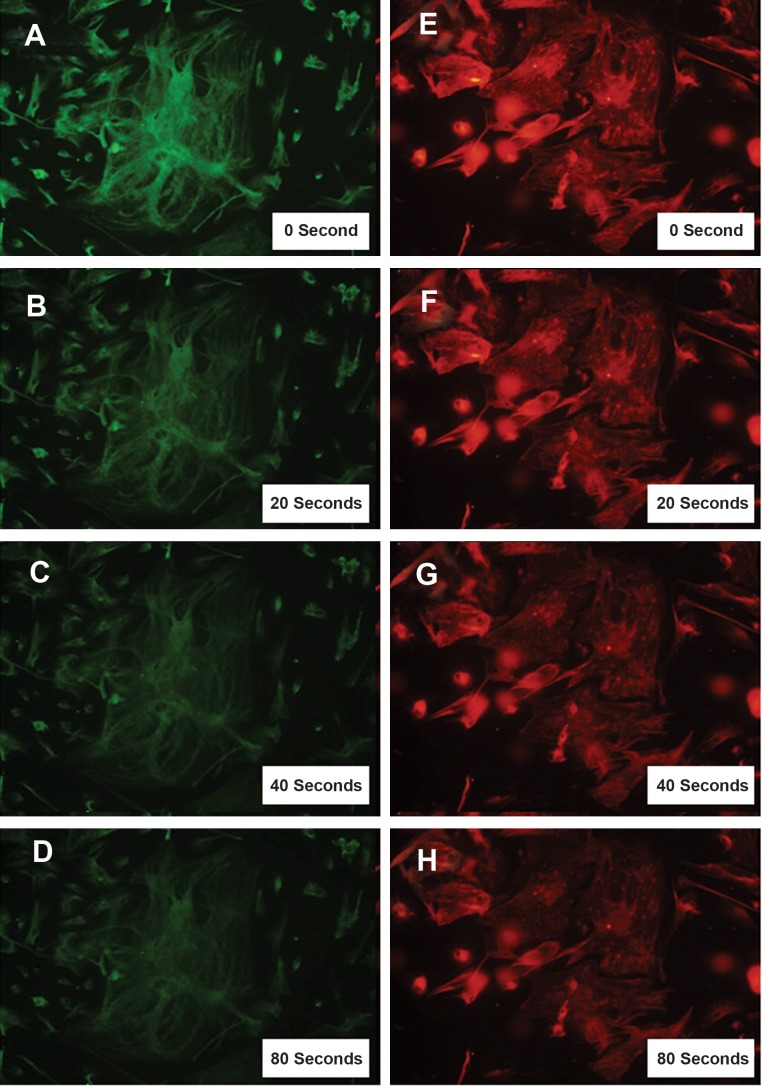
Comparison of the fluorescence of FITC- and Alexa Fluor 568- conjugated ANM using ICC staining of bovine sertoli cells.

 In order to compare the photostability of FITC- and Alexa Fluor 568- conjugated ANM, their respective fluorescent kinetics were compared. Images that were taken every 0, 20, 40, and 80 seconds revealed that Alexa Fluor 568 had more photostability than FITC (Fig 2).

 In details, images of FITC conjugated ANM (Fig 2 A, B, C, D) and those of Alexa Fluor 568 conjugated ANM (Fig 2 E, F, G, H) were continuously taken.

 Pair wise comparison of images showed an earlier quenching of FITC stained samples than those stained with Alexa Fluor 568.

**Fig 3:**
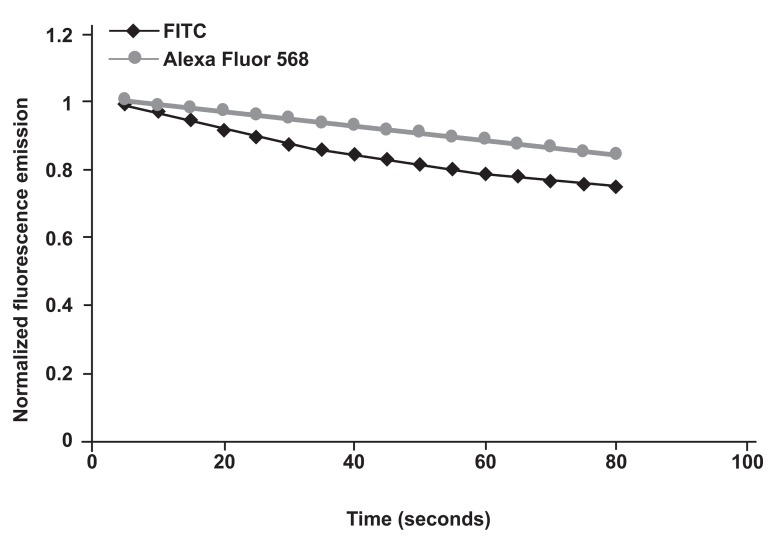
Analysis of the photostability of FITC- and Alexa Fluor 568-conjugated ANM by continuous illumination of ICC staining of bovine sertoli cells. Images were captured every 5 seconds followed by analysis of the data by ImageJ software.

 In addition, 5 seconds digital images taken of ICC stained sertoli cells were analyzed by ImageJ software. Normalized dye fluorescent intensities showed a lower photostability for FITC than that of Alexa Fluor 568 (Fig 3). Moreover, student's t-test of 5 seconds digital images processed by ImageJ software revealed significant difference between FITC and Alexa Fluor 568 (p= 0.004).

## Conclusion

 Since dye photostability and brightness are applicable traits, scientists try to compare fluorescent dyes to discover more useful ones (3, 4). At this point of view, the photostability and brightness of FITC have been compared with other dyes (7). It is presumed that the Alexa Fluor family has more fluorescent intensity and brightness in contrast to Cy dyes (8). Since researchers have reported many obstacles when working with traditional dyes such as FITC, we compared the photostability and photobleaching characteristics of FITC and Alexa Fluor 568 (5, 6, 8, 14). Our study showed that Alexa Fluor 568 had brighter fluorescence and more photostability than FITC.

 Since previous reports have suggested that 5-10 moles of Alexa Fluor 568 and 5-9 moles of FITC are attached to 1 mole of IgG in optimum molar ratios (8, 13), in our experiment, 9 moles of Alexa Fluor 568 and 7 moles of FITC were conjugated to each mole of ANM. This optimum fluorofore/antibody molar ratio ensured the conjugate's best activity.

## Conclusion

 In contrast to FITC which has restrictive and limited application in photostaining techniques, Alexa Fluor 568 has brighter fluorescence and greater photostability.
